# Evaluation of hydroxyurea genotoxicity in patients with sickle cell disease

**DOI:** 10.31744/einstein_journal/2019AO4742

**Published:** 2019-09-04

**Authors:** Emanuel Almeida Moreira de Oliveira, Kenia de Assis Boy, Ana Paula Pinho Santos, Carla da Silva Machado, Cibele Velloso-Rodrigues, Pâmela Souza Almeida Silva Gerheim, Leonardo Meneghin Mendonça

**Affiliations:** 1 Universidade Federal de Juiz de Fora, Juiz de Fora, MG, Brazil.; 2 Hemocentro Regional de Governador Valadares, Fundação Hemominas, Governador Valadares, MG, Brazil.

**Keywords:** Anemia, sickle cell, Hydroxyurea, Genotoxicity, Mutagenicity tests, Micronucleus tests

## Abstract

**Objective:**

To evaluate the induction of DNA damage in peripheral blood mononuclear cells of patients with sickle cell disease, SS and SC genotypes, treated with hydroxyurea.

**Methods:**

The study subjects were divided into two groups: one group of 22 patients with sickle cell disease, SS and SC genotypes, treated with hydroxyurea, and a Control Group composed of 24 patients with sickle cell disease who were not treated with hydroxyurea. Peripheral blood samples were submitted to peripheral blood mononuclear cell isolation to assess genotoxicity by the cytokinesis-block micronucleus cytome assay, in which DNA damage biomarkers – micronuclei, nucleoplasmic bridges and nuclear buds - were counted.

**Results:**

Patients with sickle cell disease treated with hydroxyurea had a mean age of 25.4 years, whereas patients with sickle cell disease not treated with hydroxyurea had a mean age of 17.6 years. The mean dose of hydroxyurea used by the patients was 12.8mg/kg/day, for a mean period of 44 months. The mean micronucleus frequency per 1,000 cells of 8.591±1.568 was observed in the Hydroxyurea Group and 10.040±1.003 in the Control Group. The mean frequency of nucleoplasmic bridges per 1,000 cells and nuclear buds per 1,000 cells for the hydroxyurea and Control Groups were 0.4545±0.1707 *versus* 0.5833±0.2078, and 0.8182±0.2430 *versus* 0.9583±0.1853, respectively. There was no statistically significant difference between groups.

**Conclusion:**

In the study population, patients with sickle cell disease treated with the standard dose of hydroxyurea treatment did not show evidence of DNA damage induction.

## INTRODUCTION

Sickle cell disease (SCD) is chronic, with a recessive autosomal pattern, characterized by a point mutation on the gene encoding beta-globin, one of the polypeptide chains of hemoglobin (Hb), which then is denominated hemoglobin S (HbS). This mutation triggers the loss of electrical charges, favoring the polymerization of HbS, conferring fragility and the aspect of a sickle to the red blood cells.^1^ Patients with SCD can present with various complications, such as hemolytic anemia, greater susceptibility to infections, painful crises, cardiac, hepatobiliary, ophthalmologic, osteoarticular, urogenital, pulmonary, and neurologic complications, besides severe vaso-occlusive crisis.^1 , 2^

Hydroxyurea (HU) is a drug that inhibits ribonucleotide reductase, approved in 1967 by the Food and Drug Administration (FDA) for the treatment of neoplastic diseases, and in the 1980s, was incorporated as part of the therapy for patients with SCD. The use of HU is considered a significant pharmacologic advancement in the treatment of SCD, presenting with positive results with a reduction in the number of deaths/complications and improvement in the patients’ quality of life.^3^ Its beneficial effects in the treatment of SCD include the increased concentration of fetal hemoglobin (HbF) in the red blood cells and improved metabolism of nitric oxide, reducing endothelial interaction, pain episodes, acute thoracic syndrome, hospital admissions, and need for blood transfusions.^4 , 5^

Nevertheless, treatment with HU can lead to known adverse reactions, albeit reversible, such as low neutrophil count, low platelet count, anemia, rash, headache, and occasionally, nausea. There is also evidence as to the long-term risks of treatment with HU, including its effects on fertility and on reproduction.^3^

Hydroxyurea is also known as a genotoxic agent in several *in vitro* and *in vivo* trials,^6^ leading to a concern as to possible genotoxic damages with mutagenic effects, and increased risk of malignant modifications. Thus, patient monitoring can be used with methodologies for DNA damage evaluation, in order to better understand the genotoxicity of this drug in treatment of SCD.^7^

Assessment of DNA damage can be investigated by the cytokinesis block micronucleus cytome assay (CBMN-cyt) in peripheral blood mononuclear cells (PBMC), which is a widely used method for genotoxicity research. In this study, one can quantify the formation of micronuclei (MN), which are a type of chromosome damage marker that originates from fragments of chromosomes or whole chromosomes. Additionally, nuclear buds (NBUDs), biomarkers of gene amplification, and nucleoplasmic bridges (NPBs), which frequently correspond to dicentric chromosomes, can also be evaluated.^8^

## OBJECTIVE

To evaluate the induction of DNA damage by means of cytokinesis block micronucleus cytome assay in peripheral blood mononuclear cells from patients with sickle cell disease SS and SC treated with hydroxyurea.

## METHODS

### Selection of research subjects

The present study was carried out during the period from August 2015 to January 2018. Patients with SCD seen at the *Hemocentro Regional de Governador Valadares da Fundação Hemominas* , in the city of Governador Valadares, state of Minas Gerais, were invited to participate. The area of coverage of this blood transfusion center includes the regions of the *Vales do Aço, Rio Doce, Mucuri, Jequitinhonha,* and part of *Zona da Mata.* Patients were included in the study after giving their consent by signing the Informed Consent Form (ICF) and/or the Agreement Form (AF), whenever appropriate. Pregnant patients, those who chronically used other medications besides HU, those with positive serology tests for HIV, hepatitis B and C, or who presented with severe hepatic or renal impairment, were excluded, in addition to patients who received blood transfusions within a period of less than 100 days.

Research subjects were divided into two groups; in that, one had 22 patients with SCD genotypes SS and SC, treated with HU (HU Group) and the Control Group, composed of 24 patients with SCD who were not treated with this drug.

The study followed the protocol approved by the Research Ethics Committee of the *Universidade Federal de Juiz de Fora* (CAAE: 29058814.4.0000.5147, process number 718.344).

### Collection of peripheral blood mononuclear cells

From each individual, approximately 5mL of venous blood were withdrawn in tubes containing the anticoagulant EDTA, and then the mononuclear cells were separated and added to conical tubes with 4mL Histopaque^®^-1077 (Sigma-Aldrich^®^) and 4mL of whole blood, centrifuged at 400×g, for 30 minutes, at 25º C. The interface range containing mononuclear cells was washed with phosphate buffered saline solution (PBS), and then collected after 10 minutes of centrifugation at 250×g. The resulting pellets were once again suspended in RPMI 1640 medium and transferred to cell culture flasks at the density of 1×10^5^ cells/mL.

### Evaluation da genotoxicity

The evaluation of genotoxicity was performed by a CBMN-cyt assay, carried out according to the Fenech protocol,^9^ will small modifications. The PBMC cells were cultivated in culture flask containing RPMI medium 1640, 20% bovine fetal serum, 0.6% of phytohemagglutinin A (Sigma-Aldrich^®^), 1% of L-glutamine (Sigma-Aldrich^®^), and 1% of penicillin-streptomycin (Gibco^®^), incubated in an oven at a 37°C with 5% carbon dioxide. After 44 hours of incubation, cytochalasin B (Sigma-Aldrich^®^) was added at 6.0µg/mL concentration, maintaining the cultures incubated for another 28 hours.

After the incubation time, cell collection was performed, transferring the cell suspension to conical tubes and fixating the cells in 3:1 methanol/acetic acid, and adding a hypotonic treatment in a 0.01% citrate solution. The slides were prepared and stained with acridine orange (Sigma-Aldrich^®^), and analyzed under a fluorescence microscope.

One cell culture was performed for each individual, and from this culture, two slides were prepared; a total of 2,000 binuclear cells were analyzed as to the presence of DNA damage biomarkers (number of binuclear cells with MN, NPBs, and NBUDs). All research slides were analyzed by a single examiner. The results of frequency of biomarkers MN, NPBs, and NBUDs were presented by 1,000 binuclear cells. For each individual, 500 cells were analyzed and classified as mononuclear, binuclear, trinuclear and multinuclear (four or more nuclei) to determine the Nuclear Division Index (NDI).

### Statistical analysis

The data obtained were expressed as mean±standard deviation and analyzed by means of the GraphPad Prism^®^ version 6.01 statistics software, applying the paired *t* test to compare the results among the patients with SCD, SS, or SC treated with HU, and those not treated with the drug. The significance level adopted was p<0.05.

## RESULTS

The total number of patients evaluated was 46, with Group HU composed of 22 individuals with SCD (19 SS and 3 SC) treated with HU, in which 12 were male, aged between 6 and 59 years (mean 25.4 years), receiving HU orally at 7.5 to 19.5mg/kg/day (mean 12.8mg/kg/day) between 12 and 82 months (mean 44 months). The Control Group comprised 24 individuals with SCD (14 SS and 10 SC) not treated with HU, in which 15 were male, aged between 4 and 52 years (mean 17.6 years) ( Table 1 ).

**Table 1:** Age, dose used, and duration of treatment with hydroxyurea in patients with sickle cell disease

Parameter	HU Group*			Control Group*		
	n=22			n=24		
	
	Mean	Min.	Max.	Mean	Min.	Max.
Age, years	25.4	6	59	17.6	4	52
HU dose, mg/kg/day	12.8	7.5	19.5			
Time of treatment, months	44	12	82			

* HU Group corresponded to patients with SS or SC sickle cell disease treated with hydroxyurea, and the Control Group corresponded to patients with SS or SC sickle cell disease not treated with hydroxyurea. HU: hydroxyurea; Min: minimum; Max: maximum.

**Figure 1:**
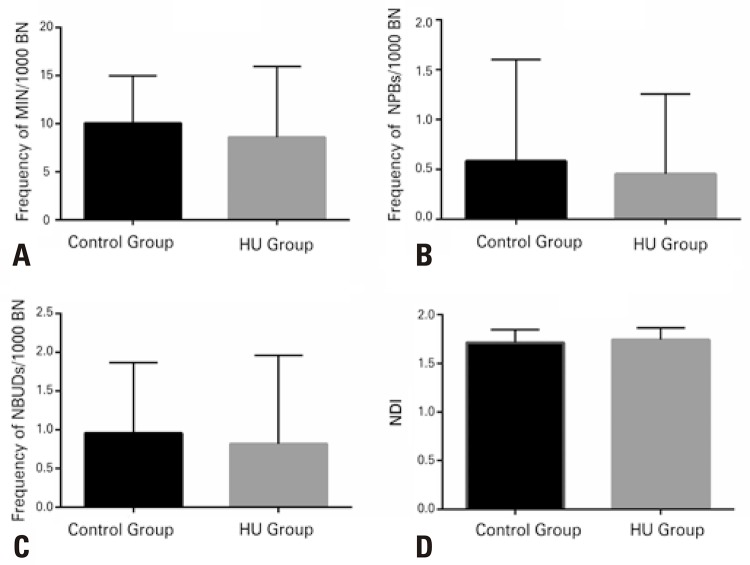
Evaluation of the genotoxicity effect by means of cytokinesis block micronucleus cytome assay in peripheral blood mononuclear cells of patients with sickle cell disease SS or SC, treated with hydroxyurea (HU Group; n=22) and not treated with this drug (Control Group; n=24). Mean frequency of micronuclei (MN): (A) nucleoplasmic bridges (NPBs); (B) nuclear buds (NBUDs); (C) and Nuclear Division Index (NDI); (D) in binuclear cells (BN). There was no significant difference between the groups studied (p>0.05), according to the paired *t* test

The DNA damage markers evaluated showed no significant differences between the groups of patients using or not HU ( Figure 1 ; all p>0.05). A mean frequency of MN per 1,000 cells was 8.591±1.568 in the HU Group and 10.04±1.003 in the Control Group. The mean frequency of NPBs per 1,000 cells for the HU Group was 0.4545±0.1707, and 0.5833±0.2078 for the Control Group. The mean of NBUDs per 1,000 cells and the NDI for the HU and Control Groups were 0.8182±0.2430 and 0.9583 *±* 0.1853, and 1.744±0.02607 and 1.932±0.02691, respectively.

## DISCUSSION

Hydroxyurea is widely used in the clinical management of patients with SCD, but the risks related to their prolonged use are still under evaluation, especially its carcinogenic potential.

The genotoxicity indicators enable assessing the effects of exposures to genetic material that lead to DNA damage, and evaluating gene mutations and chromosome damage. Some genotoxicity evaluation assays comprise chromosome aberrations, sister chromatid exchanges, reverse mutations, comet assay, and analysis of MNs, NPBs, and NBUDs. These analyses, such as the frequency of MNs, NPBs, and NBUDs, applying the CBMN-cyt methodology in human lymphocytes, can help in predictive tests regarding risk of cancer.^8 , 10^

The concerns about the carcinogenic potential of HU are due to this drug also being known as an antineoplastic agent, and there are data in literature in which patients presented with an increase in DNA damage in blood cells.^11 , 12^

There are conflicting reports as to the DNA damage potential in humans exposed to HU. Some studies showed that the substance is genotoxic, while others suggested HU has low *in vivo* mutagenicity, which emphasizes the need for research about the long-term safety of HU administration.^11 , 13 , 14^

Some studies have shown results in which there was an increase in DNA damage in blood cells of patients treated with HU in comparison with the Control Group. Friedrisch et al.,^14^ used the comet assay to analyze peripheral blood leukocytes of 28 patients with SCD treated with HU, and of 28 individuals without SCD, and they found higher levels of DNA damage in the group of patients treated with HU. Nevertheless, in this study by Friedrisch et al.,^14^ it is not possible to distinguish if the effects observed result from exposure to HU or due to the disease itself, as suggested by Rodriguez et al.,^7^ Moreover, another study presented with data in reference to 293 blood samples of 105 children, in a median of 2 years of HU therapy, in which the exposure to the drug was associated with significantly increased frequencies of MN in reticulocytes, reflecting the chromosome damage that occurred in the erythroblasts.^15^

Nonetheless, the results of the present study showed that in the population of SCD patients evaluated, there was no significant increase in DNA damage in the blood cells of patients treated with HU.

Similar to our findings, some reports in literature indicated treatment conditions in which HU did not lead to induction of DNA damage. Rodriguez et al.,^7^ employed the comet assay and found no significant difference in DNA damage between patients with SCD, treated, or not, with HU, with doses ≤30mg/kg/day. Using the CBMN-cyt assay in lymphocytes, Maluf et al.,^16^ found a small increase in the number of MN in the group of patients treated with HU, which correlated with duration of treatment and the final HU dose. In our study, in which our patients used HU doses of up to 19.5mg/kg/day, the frequency of MNs, NPBs, and NBUDs was similar between the group of patients treated with HU and the Control Group.

## CONCLUSION

The present study pointed out the safety of use of hydroxyurea for a mean period of 44 months, and in doses of up to 19mg/kg/day per patient with sickle cell disease, since there were no significant differences found in the genotoxicity markers between the group of patients with sickle cell disease using the drug or not. Although there is evidence of measurable genotoxicity due to exposure to hydroxyurea in patients, this might be related to specific situations such as elevated doses, long periods of treatment, or age range of patients.
