# Supplementary material for: Evaluation of hydroxyurea genotoxicity in patients with sickle cell disease
Source: Einstein (Sao Paulo). 2019 Sep 4;17(4):eAO4742. doi: 10.31744/einstein_journal/2019AO4742 (PMC6750882; doi:10.31744/einstein_journal/2019AO4742)
Supplement: Translation

# Translation: Avaliação da indução de genotoxicidade pela hidroxiureia em pacientes com doença falciforme

**Authors:** Emanuel Almeida Moreira de Oliveira, Kenia de Assis Boy, Ana Paula Pinho Santos, Carla da Silva Machado, Cibele Velloso-Rodrigues, Pâmela Souza Almeida Silva Gerheim, Leonardo Meneghin Mendonça

**Affiliations:** 1 Universidade Federal de Juiz de Fora, Juiz de Fora, MG, Brazil.; 2 Hemocentro Regional de Governador Valadares, Fundação Hemominas, Governador Valadares, MG, Brazil.

**Keywords:** Anemia falciforme, Hidroxiureia, Genotoxicidade, Testes de mutagenicidade, Testes para micronúcleos

## Abstract

**Objetivo:**

Avaliar o efeito da indução de danos ao DNA em células monocelulares do sangue periférico de pacientes com doença falciforme, genótipos SS e SC, tratados com hidroxiureia.

**Métodos:**

Os sujeitos da pesquisa foram divididos em dois grupos: um de 22 pacientes com doença falciforme genótipos SS e SC tratados com hidroxiureia, e o outro controle, composto por 24 pacientes com doença falciforme que não eram tratados com o fármaco. As amostras de sangue periférico foram submetidas ao isolamento de células mononucleares do sangue periférico para avaliação da genotoxicidade pelo ensaio de micronúcleo citoma com bloqueio da citocinese, tendo sido quantificados os biomarcadores de danos ao DNA – micronúcleos, pontes nucleoplasmáticas e brotamento nuclear.

**Resultados:**

Os pacientes com doença falciforme tratados com hidroxiureia apresentaram média de idade de 25,4 anos, enquanto aqueles com doença falciforme não tratados com hidroxiureia tiveram média de idade de 17,6 anos. A dose média de hidroxiureia utilizada pelos pacientes foi de 12,8mg/kg/dia, por período médio de 44 meses. A frequência média de micronúcleos por 1.000 células de 8,591±1,568 foi observada no Grupo Hidroxiureia e de 10,040±1,003 no Grupo Controle. Adicionalmente, a frequência média de pontes nucleoplasmáticas por 1.000 células e brotamento nuclear por 1.000 células para o Grupo Hidroxiureia e Controle foi de 0,4545±0,1707 *versus* 0,5833±0,2078, e de 0,8182±0,2430 *versus* 0,9583±0,1853, respectivamente. Não houve diferença estatisticamente significativa entre os grupos.

**Conclusão:**

Na população estudada de pacientes com doença falciforme com tratamento em dose padrão de hidroxiureia, não houve evidência de indução de danos ao DNA.

## INTRODUÇÃO

A doença falciforme (DF) é crônica, de transmissão autossômica recessiva, caracterizada por mutação pontual no gene, que codifica a betaglobina, uma das cadeias polipeptídicas da hemoglobina (Hb), passando a ser denominada hemoglobina S (HbS). Essa mutação desencadeia a perda de cargas elétricas, favorecendo a polimerização da HbS, conferindo fragilidade e o aspecto de foice às hemácias.^1^ Os pacientes com DF podem apresentar diversas complicações, como anemia hemolítica, maior suscetibilidade a infecções, crises dolorosas, complicações cardíacas, hepatobiliares, oftalmológicas, osteoarticulares, urogenitais, pulmonares, neurológicas e quadros graves de crises vaso-oclusivas.^1 , 2^

A hidroxiureia (HU) é um fármaco inibidor da ribonucleotídeo redutase aprovado em 1967 pela *Food and Drugs Administration* (FDA) para o tratamento de doenças neoplásicas e, na década de 1980, passou a fazer parte da terapia dos pacientes com DF. O uso da HU é considerado um avanço farmacológico importante no tratamento da DF, apresentando resultados positivos com redução no número de óbitos/complicações e melhoria na qualidade de vida dos pacientes.^3^ Seus efeitos benéficos no tratamento da DF incluem o aumento da concentração de hemoglobina fetal (HbF) nas células vermelhas do sangue e a melhora do metabolismo do óxido nítrico, reduzindo a interação endotelial, os episódios de dor, a síndrome torácica aguda, as internações hospitalares e a necessidade de transfusões sanguíneas.^4 , 5^

No entanto, o tratamento com HU pode levar a reações adversas reconhecidas, porém reversíveis, como baixa contagem de neutrófilos, baixa contagem de plaquetas, anemia, erupção cutânea, dor de cabeça e, ocasionalmente, náusea. Também há evidências sobre os riscos a longo prazo do tratamento com HU, incluindo seus efeitos na fertilidade e na reprodução.^3^

A HU também é um conhecido agente genotóxico em vários ensaios *in vitro* e *in vivo* ,^6^ levando a uma preocupação sobre possíveis danos genotóxicos com efeitos mutagênicos e aumento do risco de alterações malignas. Assim, para uma maior compreensão sobre a genotoxicidade desse fármaco quando usado em tratamento de pacientes com DF, pode ser empregado o monitoramento dos pacientes com metodologias de avaliação de danos ao DNA.^7^

A avaliação de danos ao DNA pode ser investigada pelo ensaio de micronúcleo citoma com bloqueio da citocinese (CBMN-cit) em células mononucleares do sangue periférico, sendo um método amplamente utilizado para estudos de genotoxicidade. Neste ensaio, pode-se quantificar a formação de micronúcleos (MN), que são um tipo de marcador de dano cromossômico que tem sua origem a partir de fragmentos de cromossomos ou cromossomos inteiros. Adicionalmente, brotos nucleares (NBUDs - *nuclear buds* ), biomarcadores de amplificação gênica, e pontes nucleoplásmicas (NPBs - *nucleoplasmic bridges* ), que frequentemente correspondem a cromossomos dicêntricos, também podem ser avaliados.^8^

## OBJETIVO

Avaliar a indução de danos ao DNA por meio do ensaio de micronúcleo citoma com bloqueio da citocinese em células mononucleares do sangue periférico de pacientes com doença falciforme SS e SC tratados com hidroxiureia.

## MÉTODOS

### Seleção dos sujeitos da pesquisa

O presente estudou foi realizado no período de agosto de 2015 a janeiro de 2018. Foram convidados a participar da pesquisa os pacientes com DF atendidos no Hemocentro Regional de Governador Valadares da Fundação Hemominas, em Minas Gerais. A área de abrangência do hemocentro compreende as regiões dos Vales do Aço, Rio Doce, Mucuri, Jequitinhonha e parte da Zona da Mata. Os pacientes foram incluídos na pesquisa após manifestarem o consentimento pela assinatura de Termo de Consentimento Livre e Esclarecido (TCLE) e/ou Termo de Assentimento (TA), quando apropriado. Foram excluídos da pesquisa pacientes gestantes, que faziam uso crônico de outros medicamentos, além da HU, que apresentaram testes sorológicos positivos para HIV, hepatite B e C ou que apresentaram comprometimento hepático ou renal graves, além dos pacientes que receberam transfusão sanguínea em período inferior a 100 dias.

Os sujeitos da pesquisa foram divididos em dois grupos, sendo um de 22 pacientes com DF genótipos SS e SC, tratados com HU (Grupo HU) e o Grupo Controle, composto por 24 pacientes com DF que não eram tratados com o fármaco.

A pesquisa seguiu o protocolo aprovado pelo Comitê de Ética em Pesquisa da Universidade Federal de Juiz de Fora (CAAE: 29058814.4.0000.5147, processo número 718.344).

### Obtenção de células mononucleares do sangue periférico

De cada indivíduo, foram coletados aproximadamente 5mL de sangue venoso periférico em tubos contendo EDTA como anticoagulante e, em seguida, foram separadas as células mononucleares, adicionando, em tubos cônicos, 4mL de Histopaque^®^-1077 (Sigma-Aldrich^®^) e 4mL de sangue total, centrifugando a 400×g, por 30 minutos, a 25ºC. A faixa de interface contendo células mononucleares foi lavada com solução salina tamponada com fosfato (PBS) e, a seguir, coletada após 10 minutos de centrifugação a 250×g. Os pellets resultantes foram suspensos novamente em meio RPMI 1640 e transferidos para frascos de cultura celular na densidade de 1×10^5^ células/mL.

### Avaliação da genotoxicidade

A avaliação da genotoxicidade foi realizada por meio do ensaio CBMN-cit, realizado de acordo com o protocolo de Fenech^9^ com pequenas modificações. As células mononucleares do sangue periférico foram cultivadas em frascos de cultura contendo meio RPMI 1640, 20% de soro fetal bovino, 0,6% de fitoemaglutinina A (Sigma-Aldrich^®^), 1% de L-glutamina (Sigma-Aldrich^®^) e 1% de penicilina-estreptomicina (Gibco^®^), incubadas em estufa a 37°C com 5% de dióxido de carbono. Após 44 horas de incubação, foi adicionada a citocalasina B (Sigma-Aldrich^®^) na concentração de 6,0µg/mL, mantendo as culturas incubadas por mais 28 horas.

Após o tempo de incubação foi realizada a coleta celular, transferindo a suspensão celular para tubos cônicos e fixando as células em metanol/ácido acético 3:1, e adicionando tratamento hipotônico em solução de citrato a 0,01%. As lâminas foram preparadas e coradas com laranja de acridina (Sigma-Aldrich^®^), tendo sido analisadas em microscópio de florescência.

Para cada indivíduo, uma cultura celular foi realizada e, desta cultura, duas lâminas foram confeccionadas, sendo analisadas 2.000 células binucleadas quanto a presença dos biomarcadores de danos ao DNA (número de células binucleadas com MN, NPBs e NBUDs). Todas as lâminas da pesquisa foram analisadas por um único examinador. Os resultados da frequência dos biomarcadores MN, NPBs e NBUDs foram apresentados por 1.000 células binucleadas. Para cada indivíduo, 500 células foram analisadas e classificadas como mononucleadas, binucleadas, trinucleadas e multinucleadas (quatro ou mais núcleos) para determinação do Índice de Divisão Nuclear (IDN).

### Análise estatística

Os dados obtidos foram expressos com média±desvio padrão e analisados por meio do software estatístico *GraphPad Prism* ^®^ *Version* 6.01, aplicando o teste *t* pareado, para comparar os resultados entre os pacientes com DF, SS ou SC tratados com HU, e aqueles não tratados com tal fármaco. O critério de significância utilizado foi de p<0,05.

## RESULTADOS

No total, foram avaliados 46 pacientes, com o Grupo HU composto por 22 indivíduos com DF (19 SS e 3 SC) tratados com HU, sendo 12 do sexo masculino, com idade entre 6 e 59 anos (média 25,4 anos), recebendo HU por via oral a 7,5 a 19,5mg/kg/dia (média de 12,8mg/kg/dia) entre 12 e 82 meses (média de 44 meses). O Grupo Controle foi composto por 24 indivíduos com DF (14 SS e 10 SC) não tratados com HU, sendo 15 do sexo masculino, com idade entre 4 e 52 anos (média 17,6 anos) ( Tabela 1 ).

**Tabela 1:** Idade, dose utilizada e tempo de tratamento com hidroxiureia nos pacientes com doença falciforme

Parâmetro	Grupo HU*			Grupo Controle*		
	n=22			n=24		
	
	Média	Min.	Max.	Média	Min.	Max.
Idade, anos	25,4	6	59	17,6	4	52
Dose de HU, mg/kg/dia	12,8	7,5	19,5			
Tempo de tratamento, meses	44	12	82			

* Grupo HU correspondeu a pacientes com doença falciforme SS ou SC tratados com hidroxiureia, e Grupo Controle correspondeu a pacientes com doença falciforme SS ou SC não tratados com hidroxiureia. HU: hidroxiureia; Min: mínimo; Max: máximo.

Os marcadores de danos ao DNA avaliados não apresentaram diferenças significativas entre o grupo de pacientes usando HU e aqueles sem o uso do fármaco ( Figura 1 ; todos p>0,05). Obteve-se frequência média de MN por 1.000 células igual a 8,591±1,568 no Grupo HU e de 10,04±1,003 no Grupo Controle. A frequência média de NPBs por 1.000 células para o Grupo HU foi de 0,4545±0,1707 e 0,5833±0,2078 para o Controle. A média observada dos NBUDs por 1.000 células e o IDN para os Grupos HU e Controle foram 0,8182±0,2430 e 0,9583 *±* 0,1853, e 1,744±0,02607 e 1,932±0,02691, respectivamente.

**Figura 1:** Avaliação do efeito de genotoxicidade por meio do ensaio de micronúcleo citoma com bloqueio da citocinese em células mononucleares de sangue periférico de pacientes com doença falciforme SS ou SC tratados com hidroxiureia (Grupo HU; n=22) e não tratados com esse fármaco (Grupo Controle; n=24). Frequência média de micronúcleos (MN): (A) pontes nucleoplasmáticas (NPBs); (B) brotos nucleares (NBUDs); (C) e Índice de Divisão Nuclear (IDN); (D) em células binucleadas (BN). Não houve diferença significativa entre os grupos estudados (p>0,05) de acordo com o teste *t* pareado

## DISCUSSÃO

A HU é amplamente utilizada no manejo clínico de pacientes com DF, mas os riscos relacionados ao seu uso prolongado ainda estão sendo avaliados, especialmente seu potencial carcinogênico.

Os indicadores de genotoxicidade permitem avaliar os efeitos de exposições ao material genético que levem à lesão no DNA, e à avaliação de mutações gênicas e danos cromossômicos. Alguns ensaios de avaliação de genotoxicidade compreendem aberrações cromossômicas, troca de cromátides irmãs, mutações reversas, ensaio cometa, e análise de MNs, NPBs e NBUDs. Essas análises, como a da frequência de MNs, NPBs, NBUDs, aplicando a metodologia CBMN-cit em linfócitos humanos, pode auxiliar em exames preditivos para risco de câncer.^8 , 10^

As preocupações sobre o potencial carcinogênico da HU devem-se a este fármaco também ser um conhecido agente antineoplásico, e há dados da literatura em que os pacientes apresentaram aumento de danos ao DNA em células sanguíneas.^11 , 12^

Existem relatos conflitantes sobre o potencial de dano ao DNA em humanos expostos à HU. Alguns estudos mostraram que a substância é genotóxica, enquanto outros sugerem que a HU tem baixa mutagenicidade *in vivo* , o que enfatiza a necessidade de pesquisas sobre a segurança a longo prazo da administração da HU.^11 , 13 , 14^

Alguns estudos mostraram resultados em que houve aumento de danos ao DNA em células sanguíneas de pacientes tratados com a HU em comparação com o Grupo Controle. Friedrisch et al.,^14^ usaram o ensaio cometa para analisar leucócitos do sangue periférico de 28 pacientes com DF tratados com HU, e de 28 indivíduos sem DF, e encontraram maiores níveis de danos no DNA no grupo de pacientes tratados com HU. Entretanto, neste estudo de Friedrisch et al.,^14^ não é possível distinguir se os efeitos observados são decorrentes da exposição à HU ou decorrentes da própria doença, como sugerido por Rodriguez et al.^7^ Adicionalmente, outro estudo apresentou dados referentes a 293 amostras de sangue de 105 crianças, em uma mediana de 2 anos de terapia com HU, na qual a exposição ao fármaco foi associada a frequências significativamente aumentadas de MN em reticulócitos, que refletem os danos cromossômicos que ocorreram nos eritroblastos.^15^

Entretanto, os resultados do presente estudo mostraram que na população de pacientes com DF avaliada não houve aumento significativo de danos ao DNA nas células sanguíneas dos pacientes tratados com a HU.

Semelhante aos nossos achados, alguns relatos na literatura indicam condições de tratamento em que a HU não levou à indução de danos ao DNA. Rodriguez et al.,^7^ utilizando teste de cometa, não encontraram diferença significativa nos danos ao DNA entre pacientes com DF, tratados ou não tratados com HU, em doses ≤30mg/kg/dia. Utilizando o ensaio do CBMN-cit em linfócitos, Maluf et al.,^16^ encontraram pequeno aumento no número de MN no grupo de pacientes tratados com HU, correlacionado com o tempo do tratamento e a dose final de HU. Em nosso estudo, em que os pacientes utilizaram doses de HU de até 19,5mg/kg/dia, a frequência de MNs, NPBs, NBUDs foi semelhante entre o grupo de pacientes tratados com HU e o Grupo Controle.

## CONCLUSÃO

O presente estudo apontou para a segurança do uso da hidroxiureia em um período médio de 44 meses, e em doses de até 19mg/kg/dia por pacientes com doença falciforme, uma vez que não foram encontradas diferenças significativas nos marcadores de genotoxicidade entre o grupo de pacientes com doença falciforme usando ou não o fármaco. Embora existam evidências de genotoxicidade mensurável devido à exposição à hidroxiureia em pacientes, esta pode estar relacionada a situações específicas como doses elevadas, longos períodos de tratamento ou faixa etária dos pacientes.
